# Discounting multiple benefits of cat containment by reframing them as trade-offs: Response to Glanville *et al.* (2025)

**DOI:** 10.1017/awf.2026.10074

**Published:** 2026-04-21

**Authors:** Sarah Legge, John Woinarski, Christopher Dickman, Tida Nou, Jaana Dielenberg

**Affiliations:** 1 Charles Darwin University, Australia; 2 Australian National University, Australia; 3 University of Sydney, Australia; 4 PO Box 15187, City East QLD 4002, Australia; 5 University of Melbourne Department of Zoology, Australia

**Keywords:** biodiversity conservation, free-roaming cats, cat containment, welfare

## Abstract

Domestic cats (*Felis catus*) are favoured companion animals, but also highly effective predators that have caused substantial declines and extinctions of native fauna in many places where they have been introduced. In Australia, free-roaming pet cats kill hundreds of millions of native animals annually, contributing to one of the world’s most severe modern extinction crises. In response, policy, advocacy and public messaging has increasingly promoted responsible pet cat management, including containment, on the multiple grounds of biodiversity protection, public amenity, disease spread reduction, and benefits to cat health and welfare. A recent article by Glanville C, Hampton JO, and Sandøe P 2025 Calling a trade-off a trade-off in arguments for cat confinement. Animal Welfare 34: e65. https://doi.org/10.1017/awf.2025.10041. challenges this messaging, arguing that claiming welfare benefits from cat containment is wilfully or negligently misleading. Here, we respond to four central arguments advanced by Glanville *et al.* We argue that separating physical health from welfare is conceptually flawed; that containment, like free-roaming, involves welfare pros and cons to cats (and their prey) that must all be evaluated concurrently; that privileging a cat’s freedom to roam ignores the welfare and rights of the animals harmed by roaming cats; and that allegations of “*bad faith*” or deceptive messaging by containment advocates are unfounded and unhelpful. While acknowledging that containment requires enriched care from cat owners, any uncertainty regarding relative welfare outcomes of containment versus free-roaming for cats does not justify ignoring the clear and severe welfare and conservation harms imposed on other animals by free-roaming cats. In Australia, pet cat containment remains a pragmatic pathway that aligns conservation objectives with overall animal welfare.

## Introduction

Cats (*Felis catus*) were domesticated in North Africa and the Near East, then dispersed by people to every inhabited continent and very many islands (De Martino *et al.* 2025). Cats can make excellent pets. They are also superlative, generalist hunters; when cats are introduced to areas that lack any native small felid, the indigenous fauna are evolutionarily naïve, and population declines and extinctions frequently result. This has played out on many small islands globally (Van Aarde & Skinner 1981; Fitzgerald & Veitch 1985; Medina *et al.* 2011), and also on larger landmasses with a unique, isolated history such as Australia (Woinarski *et al.* 2019). Roughly 40 species of endemic mammal have been lost since Europeans brought cats to Australia; in most of these cases, cats were the primary or one of the primary drivers for that loss (Woinarski *et al.* 2015, 2026; Legge *et al.* 2023).

Over the past 20 or so years, the science, management and policy sectors in Australia have collaborated impressively to gather the evidence base for cat ecology and impacts, and to use that to inform management options for both feral and pet cats (Legge *et al.* 2020c). Improving pet cat (as well as feral cat) management is necessary because free-roaming pet cats kill very large numbers of native animals (Legge *et al.* 2020d), they spread cat-dependent parasitic pathogens that affect wildlife, people and livestock (Legge *et al.* 2020a), they can be a serious nuisance to neighbours and in public areas, and they can be a source for feral cat populations (Department of Climate Change, Energy, the Environment and Water [DCCEEW] 2024).

In Australia, advocacy has encouraged policy and law reform that improves pet cat management by: requiring cat registration and identification; desexing; caps on the number of pet cats in a household; and recommending containment of pet cats to the owner’s property, ideally indoors with access to an enclosed outdoor cat-run (e.g. Legge *et al.* 2020b; Biodiversity Council 2024). The public messaging for this suite of responsible pet ownership actions, including containment, has focused on the benefits to other animals (that are not maimed or killed by the cat), as well as the cat’s health and welfare, and on reducing the disease risk for the pet-owning family (Invasive Species Council [ISC] *et al.* 2023; Royal Society for the Prevention of Cruelty to Animals [RSPCA] 2025; WA Feral Cat Working Group 2025).

In a recent article, Glanville *et al.* (2025) criticised the public messaging in Australia relating to cat containment. The article does not dispute the biodiversity benefits from containment but focuses on the messaging ‘sub-clause’ that an indoor lifestyle benefits the welfare of pet cats. The authors argue against this claim, making four main points to which we respond here.

### Sickness and injury risks for outdoor cats are health, not welfare, issues

First, Glanville *et al.* claim that this public message conflates the more limited concept of cat physical health with cat welfare. They argue that indoor cats may live longer, safer lives, but that their ability to express some of their natural behaviours is curtailed, and this might lead to stress, frustration, weight gain and various other lifestyle-related diseases – i.e. there may be detrimental consequences as well as benefits. The authors conclude that cat containment advocates who mention cat welfare benefits have “*a narrow and outdated*” or “*antiquated and reductionist*” view of welfare, focused on “*safety and physical health*”.

However, separating health from other aspects of a cat’s well-being seems myopic in its own way: as Glanville *et al.* accede, there is copious evidence that outdoor pet cats are more exposed to parasitic diseases, vehicle strikes, toxins, attacks from domestic dogs and other large animals, and persecution from people (Chalkowski *et al.* 2019; Tan *et al.* 2020). The increase in risks from an outdoor lifestyle is substantial: for example, in a survey involving 5,383 Australian cat owners, Elliot *et al.* (2019) found that 66.3% of cat owners reported losing at least one cat to a roaming-related incident, most often car strike. These are not simply issues of physical health; broken legs, ruptured abdomens, repeated trips to the vet, and becoming lost all run the risk of causing extreme distress, chronic stress, and constraining or preventing a cat’s behaviour in myriad ways.

### An indoor life is bad for the cat

Glanville *et al.* claim that an enforced, contained lifestyle poses risks to a cat’s welfare. The paper concedes that there “*are both welfare benefits and risks for cats associated with either confinement or free-roaming*” but then sweeps that complexity aside to claim that containment advocates are only concerned about “*lifespan*”, without any supporting evidence. Glanville *et al.* fall victim to the criticism they level at others, reducing the balance sheet of the welfare pros and cons to a single variable, ‘lifespan’, without properly considering the welfare advantages of a contained life, or the welfare advantages and disadvantages of an outdoor life. Indeed, picking the lifestyle with the largest net welfare benefit to the cat is not easy, precisely because there are pros and cons to both options, and reviews and meta-analyses of this question tend to conclude ‘it depends’ (Foreman-Worsley & Farnworth 2019; Tan *et al.* 2020), in part influenced by the owner’s capacity and interest in responding to cat concerns (Lawson *et al.* 2020). It is worth noting that the Australian Veterinary Association, RSPCA and People for the Ethical Treatment of Animals (AVA 2022; PETA 2026; RSPCA 2025) all support pet cat containment on welfare grounds.

Glanville *et al.* opine that the conservation message is undermined when proponents of cat containment do not explicitly recognise welfare trade-offs, and consequently this “*limits the efficacy of behaviour change interventions to increase confinement*”. This seems an ill-founded point, as containment proponents have repeatedly outlined actions that help increase welfare outcomes for contained cats, and thus containment success (Zoos Vic 2018; AVA 2022; ISC 2022; PETA 2025; RSPCA 2025; Zoo and Aquarium Association [ZAA] 2025). There is abundant evidence that negative consequences of containment can be minimised (and hence the net benefit of containment to the cat increased) by a range of responses, including: selecting appropriate cat breeds when choosing pets; early socialisation; engaging in play; provision of toys and other environmental enrichment; walking the cat on a leash; diet; and appropriate provisioning of litter trays (e.g. Jongman 2007; Cisneros *et al.* 2022; Kogan *et al.* 2024).

We cannot ask an indoor cat if having the best spot on the couch, adjacent to the best scratching post (the corner of the same sofa, oops), regular meals, and a constant supply of toys and head rubs, is just too… vanilla? Nor can we ask an outdoor cat if the thrills of free-roaming offsets the spills of a shorter life, peppered with bouts of disease, electrifying near-misses or injuries from cars and dogs, vet visits for abscess treatments and so on. Perhaps the answer to that question depends upon the cat, and the owner. Perhaps also, the differences in views on this issue might reflect one’s own projections, as much as anything else (Foreman-Worsley *et al.* 2021; Ovenden *et al.* 2024). However, what we can say with certainty is that the consequences of the two lifestyles vary enormously for other animals: in Australia and on many smaller islands these consequences have led to species extinctions and ongoing population declines of native animals. Uncertainty regarding relative welfare outcomes does not justify ignoring these well-established harms.

### Cats have rights, but other animals do not

These consequences of cat lifestyles for other animals lead us to the third point made by Glanville *et al.* The authors dwell on the ethics of allowing pet cats to make their own choices, implying that responsible pet-owners should achieve this by letting their pet cats roam, because containment is a “*clear deprivation of natural behaviour*”. However, free-roaming is not the single factor upon which a cat’s agency and natural behaviour hinges: contained cats are able to make a range of choices and express many natural behaviours.

More importantly, Glanville *et al.*’s position assumes that rights belong only to cats, ignoring that unfettered roaming of pet cats clearly has detrimental consequences to the rights and welfare of many other animals. A wounded bird being played with by a roaming cat has lost the ability to express any natural behaviour other than unconstrained terror and pain, and their welfare has been compromised. Over 300 million native birds, mammals and reptiles that are killed by roaming pet cats in Australia each year (Legge *et al.* 2020d; ISC *et al.* 2023) and many more native animals are affected by the ‘landscape-of-fear’, in which the presence of roaming cats induces a stress response and affects behaviour and welfare (Fardell *et al.* 2020). Such inevitable consequences of roaming by pet cats are ignored by Glanville *et al.* in their narrowly focused assessment of the animal welfare implications of cat containment.

If the welfare outcomes to a cat from containment are uncertain, and/or can be addressed with enrichment, whilst the welfare outcomes for many other animals from cat containment are unreservedly positive, then the best course of action seems clear, unless one considers the cat’s rights to roam should somehow trump everything else.

### Shoot the messenger

Glanville *et al.* make a series of statements that are excessive and seem designed to offend other scientists working in this space, rather than to make a sensible case. This unnecessary colouring of the argument is evident even in their terminology; notably labelling containment as a “*tyranny*”, a term that implicitly loads guilt on cat-owners who responsibly manage their pet cats.

In a *reductio ad absurdum* argument, Glanville *et al.* explicitly suggest a moral equivalence: that advocating for the confinement of pet cats is comparable to condoning battery cages for hens, farrowing pens for pigs, and cages for zoo animals rather than open-range settings. Equating the experience of a much-loved, contained cat with the abject misery of a battery hen’s life is inappropriate, and offensive to containment advocates, as well as the many, many responsible pet owners who devote copious love and care to their contained cats.

Glanville *et al.* suggest that the authors of published papers that have described cat containment as a “*win-win for wildlife and cats*” are being duplicitous. The put-downs pepper their paper: they claim that advocates for cat containment are being “*disingenuous*”, making a “*dubious assertion*”; that they are spreading a “*bad faith*” message; engaging in “*welfare-washing*” and “*insincere appeals*”; being “*downright deceptive*”; and telling “*white lies*”. The citation used by Glanville *et al.* as the parallel for repercussive dangers from telling white lies is a paper that reports on a recent, highly publicised series of faked records of the critically endangered night parrot (*Pezoporus occidentalis*) in Australia. Glanville *et.al* therefore equate advocating for evidence-based policy and law reform so that pet cats are contained, in order to prevent wildlife trauma, population declines and wildlife extinctions, while also improving the health and welfare of pet cats, as comparable to serious scientific fraud. This argued guilt by association is an assertion as offensive as it is baseless.

Glanville *et al.* also state that advocates for cat containment “*either lack knowledge of the tenets of contemporary animal welfare science or are deliberately misleading the public for political reasons*”; this is a set of claims that seems patronising and defamatory at the same time. We are authors of some of the papers that are targets of this criticism, and we emphatically reject the assertion that we set out to deceive. We stand by our representation of cat containment as one of the rare solutions to a complex puzzle involving potentially competing conservation and welfare agendas. Glanville *et al.* claim that all these “*bad faith*” (etc) messages will erode public trust in science. We suggest that articles that stoop to assertions that researchers are being “*downright deceptive*” and telling “*white lies*” are more likely to erode, or destroy, trust.

### What’s really the main game here?

The hyperbole in Glanville *et al.* strikes us as a pity because the authors make important points that are clearly true. For example, they suggest that it is better that we simply choose not to have a pet cat, as it removes issues for wildlife, cat rights and welfare. Glanville *et al.* also recognise that cats do affect biodiversity, and claim that they are not opposed to containment *per se.* But from this point they veer off the track, by stating that conversations about pet cat management should focus on the risks of containment. One may as well debate the choice of font on book covers whilst standing in the middle of a burning library. Yes, containment will require cat owners to change their approach to cat care, to be more responsive, empathetic and responsible. Solving the risks of confinement to a cat is highly possible as described, for example, by Lawson *et al.* (2020). The solution to potential detriment for a contained cat is to enrich the environment, and cat owners can be supported with information on how best to achieve that. Glanville *et al.* themselves state that cat owners can mitigate or overcome any ill-effects of containment. In contrast, the task of preventing more mammal extinctions in Australia, or to island fauna generally… this is the stuff of nightmares, and Australia’s native mammals do not have time to wait whilst we debate the font size.

The issue of pet cat management tends to invite discourse that is infused with our value systems, and crosses fields with frameworks that can seem immiscible; ethics, animal rights, welfare, human well-being, biodiversity conservation. However, it is possible to weigh up outcomes of alternative actions across these frameworks, and find pathways that aim for a reasonable balance given the context (e.g. Nilsen *et al.* 2023). In Australia, the biodiversity costs from free-roaming cats are horrendous, overall animal rights and welfare are optimised when cats are not roaming, any welfare risks to cat containment can be adequately addressed, and banning cats as pets is not happening any time soon. So, the pathway is clear. Please, can we get on with the task of getting our pet cats (happily) contained?
